# The interaction of human immunodeficiency virus-1 and human endogenous retroviruses in patients (primary cell cultures) and cell line models

**DOI:** 10.1128/spectrum.01379-23

**Published:** 2023-10-09

**Authors:** Federica Mantovani, Konstantina Kitsou, Dimitrios Paraskevis, Pagona Lagiou, Gkikas Magiorkinis

**Affiliations:** 1 Department of Hygiene, Epidemiology and Medical Statistics, Medical School, National and Kapodistrian University of Athens, Athens, Greece; 2 Department of Epidemiology, Harvard T. H. Chan School of Public Health, Boston, Massachusetts, USA; Kumamoto Daigaku, Kumamoto, Kumamoto, Japan

**Keywords:** human endogenous retroviruses, HERV, HIV, retroviruses, intra-viral interactions

## Abstract

Human endogenous retroviruses (HERVs) integrated into the human genome millions of years ago, and their expression has been described both in healthy tissues and in pathology. In the present study, we study the differential expression of multiple HERV families in the presence of human immunodeficiency virus (HIV) infection in the peripheral blood mononuclear cells (PBMCs) of HIV-positive individuals, in HIV-infected cell lines, and *in vitro* HIV-infected macrophages/monocytes of healthy donors, utilizing publicly available data sets for secondary analysis using a pipeline appropriate for HERV transcription detection and comparison. We recognized significantly decreased expression of HERV-H in treatment-naïve HIV patients with viremia and increased expression of multiple HERV families in the PBMCs of HIV-infected individuals under combination antiretroviral therapy compared to healthy donors, in *in vitro* HIV-infected Stanford University pediatric T (SUP-T1) cells at 24 hours after HIV infection compared to non-infected SUP-T1 cells, and in *in vitro* HIV-infected MT4 T cells at 3 days after HIV infection compared to non-infected MT4 T cells. We recognized significantly increased expression of HERV-W, after HIV infection in monocyte-derived macrophage cell culture; no difference was found in HERV expression after HIV infection of monocyte cultures. We found a significantly decreased expression of HERV-K (HML-6) in donor-derived macrophage cultures at 36 hours after HIV infection, which does not persist at 6 days after infection. HIV infection modifies the expression of multiple HERV families in the PBMCs of HIV patients and in T cell-derived cultures, while the effect of HIV infection on HERV expression appears to be more restricted in cells of monocytic origin.

**IMPORTANCE:**

In this work, we demonstrated that human immunodeficiency virus (HIV) infection leads to the modification of the human endogenous retrovirus (HERV) expression. Differential expression of multiple HERVs was found in peripheral blood mononuclear cells derived from HIV-infected patients compared to healthy donors and HIV-infected T cell cultures compared to non-infected. The effect of HIV presence on HERV expression appears to be more restricted in cells of monocytic origin, as only deregulation of HERV-W and HERV-K (HML-6) was found in these cell cultures after their infection with HIV. Multiple factors contribute to this aberrant HERV expression, and its levels appear to be modified in a time-dependent manner. Further studies and the development of optimized *in vitro* protocols are warranted to elucidate the interactions between HIV and HERVs in detail.

## INTRODUCTION

Human endogenous retroviruses (HERVs) are the result of retroviral integrations that took place millions of years ago and currently represent around 8% of human DNA being classified as Long terminal repeat (LTR) retrotransposons. After the accumulation of deleterious mutations and homologous recombination events, leading to the creation of “solo” LTRs (1), HERVs have lost their ability of replication and retrotransposition (2). HERVs demonstrate open reading frames with coding ability (3) and their LTRs possess promoter/enhancer functions (4). Furthermore, HERV transcription has been described in healthy tissues, and demonstrates tissue specificity, and aberrant HERV transcription has been correlated to multiple human conditions, mostly inflammatory conditions, and malignancies (5, 6).

HERV integrations are more commonly found within the euchromatic areas, as those areas are more accessible to DNA-integrase complexes (7). Nevertheless, due to negative selection, most HERV integrations are found in introns or adjacent to genes, presumably to avoid interference with host’s gene integrity and thus the host’s well-being (8). Similarly, integrations in the antisense orientation are more common in the human genome (9). During the evolutionary history, these elements became fixed into the host DNA and now are “transmitted” vertically according to Mendelian inheritance (10).

Multiple HERV families are embedded in the human genome. HERV-K family is made up of 10 groups, HML-1 to HML-10 (11). HERV-K (HML-2), hereafter HK2, is the most recent to have infected the human genome, and some of its integrations are polymorphic, i.e., these integrations are only found in proportion of the population (3, 12, 13). To maintain the host’s fitness, HERV expression is under strict control mediated by multiple mechanisms (14). Increased HK2 expression has been reported after exposure to different factors, including environmental and infectious triggers, and viral transactivation has been described (15). The implication of HERVs, and more specifically HK2, in multiple human diseases has led to HK2 products being suggested as potential disease biomarkers, more emphatically on malignant diseases (16).

Various intraviral interactions have been described between human immunodeficiency virus (HIV) and HERVs, and upregulation of multiple HERV families has been described in cell lines in the setting of HIV infection (17, 18). The interactions between HK2 and HIV have been more extensively studied, and the HIV-mediated transactivation of HERVs has been shown as well as the effects of HERV-K Gag on the reduction of HIV-1 replication, particle release, and infectivity (19). Furthermore, HERV envelope (Env) proteins have antigenic properties and thus are recognized as exogenous antigens leading to innate and adaptive immune responses, shaping the antiviral immune responses of the host (20).

In the present study, we utilized publicly available data sets for secondary analysis using a pipeline appropriate for HERV transcription detection, and we demonstrate the differential expression of multiple HERV families in the peripheral blood mononuclear cells (PBMCs) of HIV-positive individuals compared to healthy controls, in different HIV-infected cells lines compared to the uninfected ones, and in *in vitro* HIV-infected macrophages/monocytes of healthy donors compared to non-infected cells.

## RESULTS

### Significantly decreased HERV-H expression in the PBMCs of treatment-naïve HIV-infected patients with viremia compared to healthy controls

**Fig 1:**
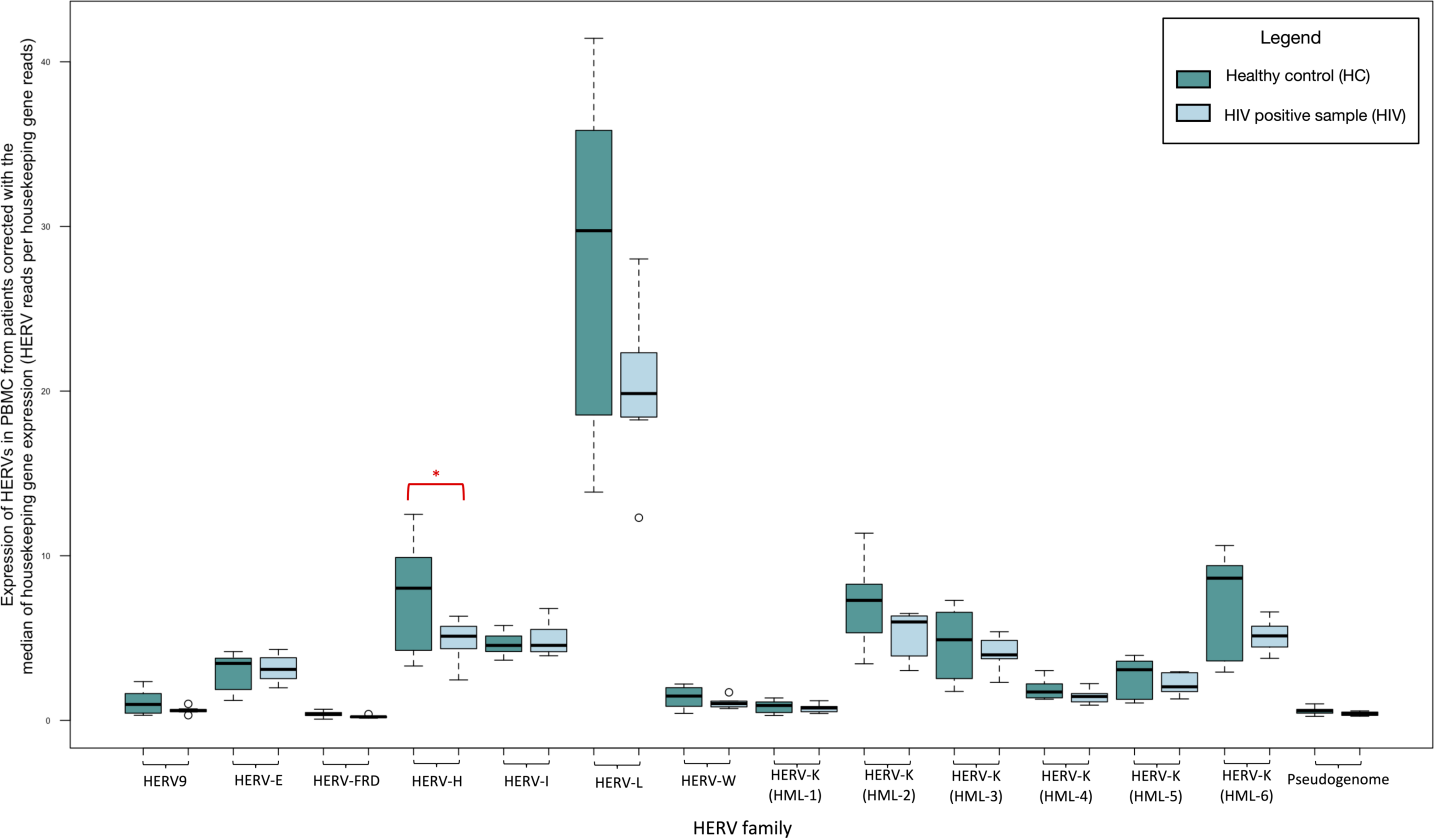
Transcription of HERVs in the PBMCs of treatment-naïve patients with HIV compared to healthy controls normalized by the median of the transcription of two housekeeping genes (succinate dehydrogenase A and hypoxanthine phosphoribosyltransferase 1) expressed as HERV reads per housekeeping gene reads. The significantly decreased expression of HERVs is highlighted with red boxes. * indicates *P* < 0.05.

We recognize a significant decrease in expression of HERV-H (fold change: 0.623, *P*-value <0.05) in the PBMCs of treatment-naïve patients compared to healthy controls. We did not recognize further differences in the expression of other HERV families nor in the HK2 integration sites (see Materials and Methods) (Fig. 1; Table S1).

### Significantly increased HERV expression in the PBMCs of HIV-infected patients under combination antiretroviral therapy (cART) compared to healthy controls

**Fig 2:**
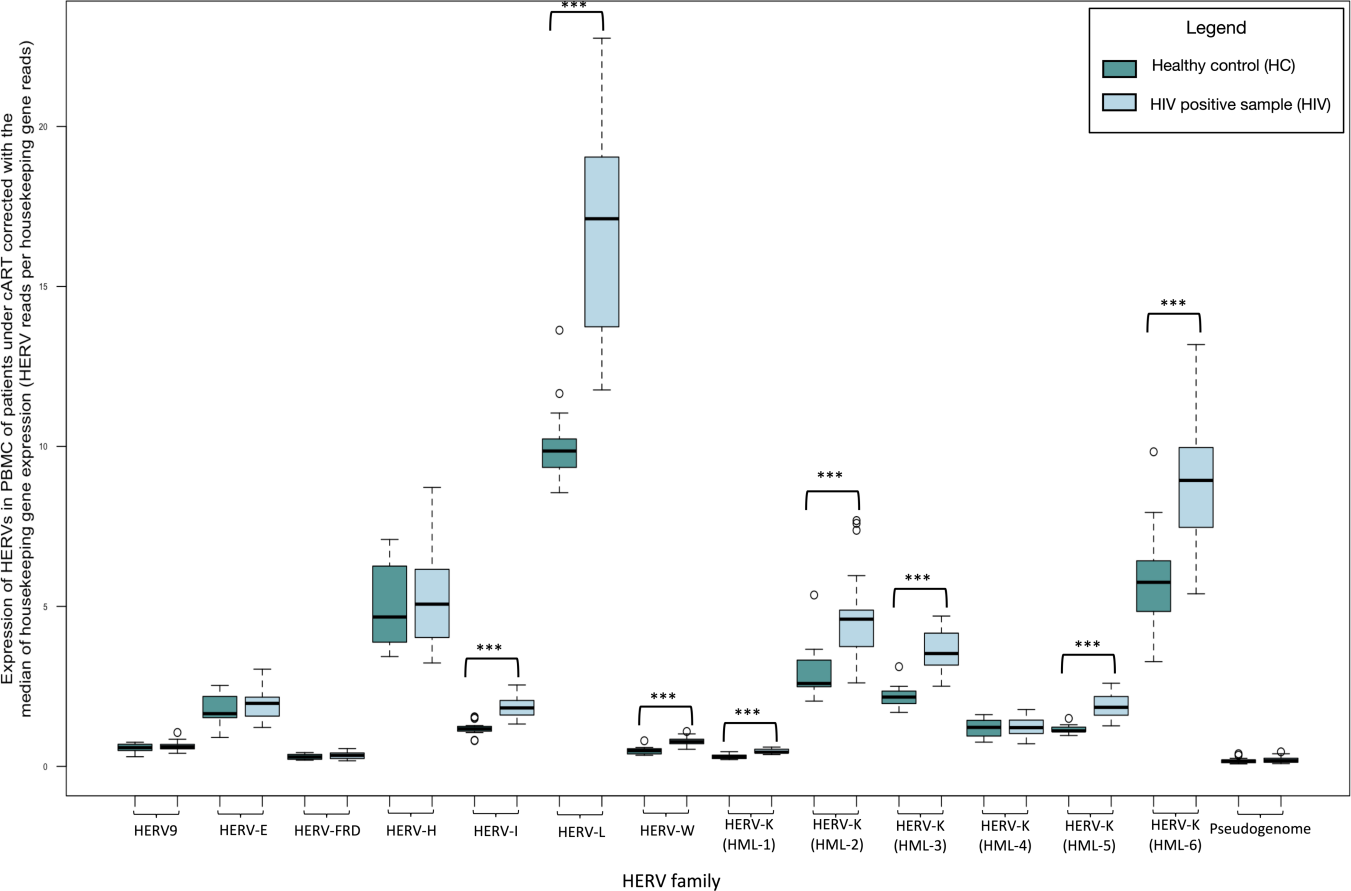
Transcription of HERVs in the PBMCs of patients with HIV undergoing cART compared to healthy controls normalized by the median of the transcription of two housekeeping genes (succinate dehydrogenase A and hypoxanthine phosphoribosyltransferase 1) expressed as HERV reads per housekeeping gene reads. The significantly increased expression of HERVs is highlighted with black boxes. *** indicate *P* < 0.001.

We recognized significantly increased expression of HERV-I (fold change: 1.538, *P*-value <0.001), HERV-L (fold change: 1.656, *P*-value <0.001), HERV-W (fold change: 1.610, *P*-value <0.001), HERV-K (HML-1) (fold change: 1.556, *P*-value <0.001), HK2 (fold change: 1.594, *P*-value <0.001), HERV-K (HML-3) (fold change: 1.645, *P*-value <0.001), HERV-K (HML-5) (fold change: 1.633, *P*-value <0.001), and HERV-K (HML-6) (fold change: 1.516, *P*-value <0.001) in the PBMCs of HIV patients under cART compared to healthy controls (Fig. 2; Table 1). Also, in order to characterize the HK2 transcription at its integration sites, we used a pseudogenome (see Materials and Methods). We did not recognize significant differences in the expression of the HK2 junction sites.

**TABLE 1:** Transcription levels of HERVs in PBMCs from patients with HIV under cART compared to PBMCs from healthy donors normalized by the median of the transcription of two housekeeping genes (SDHA and HPRT1) expressed as HERV reads per housekeeping gene reads^*a*^

	*P*-value	Ratio
HERV-9	0.301	1.082
HERV-E	0.360	1.072
HERV-FRD	0.293	1.132
HERV-H	0.789	1.023
HERV-I	**<0.001**	**1.538**
HERV-L	**<0.001**	**1.656**
HERV-W	**<0.001**	**1.610**
HML-1	**<0.001**	**1.556**
HML-2	**<0.001**	**1.594**
HML-3	**<0.001**	**1.645**
HML-4	0.576	1.033
HML-5	**<0.001**	**1.633**
HML-6	**<0.001**	**1.516**
Pseudogenome	0.524	1.616

^*a*^ The significant results are in bold. HPRT1, hypoxanthine phosphoribosyltransferase 1; SDHA, succinate dehydrogenase A. Pseudogenome: To characterize the HK2 transcription at its integration sites, we created a “pseudogenome,” based on the annotated “solo” LTR-start and -end coordinates of the HK2 sites.

### Significantly increased HERV expression in HIV LAI (strain derived from patient LAI)-infected Stanford University pediatric T (SUP-T1) cells at 24 hours

**Fig 3:**
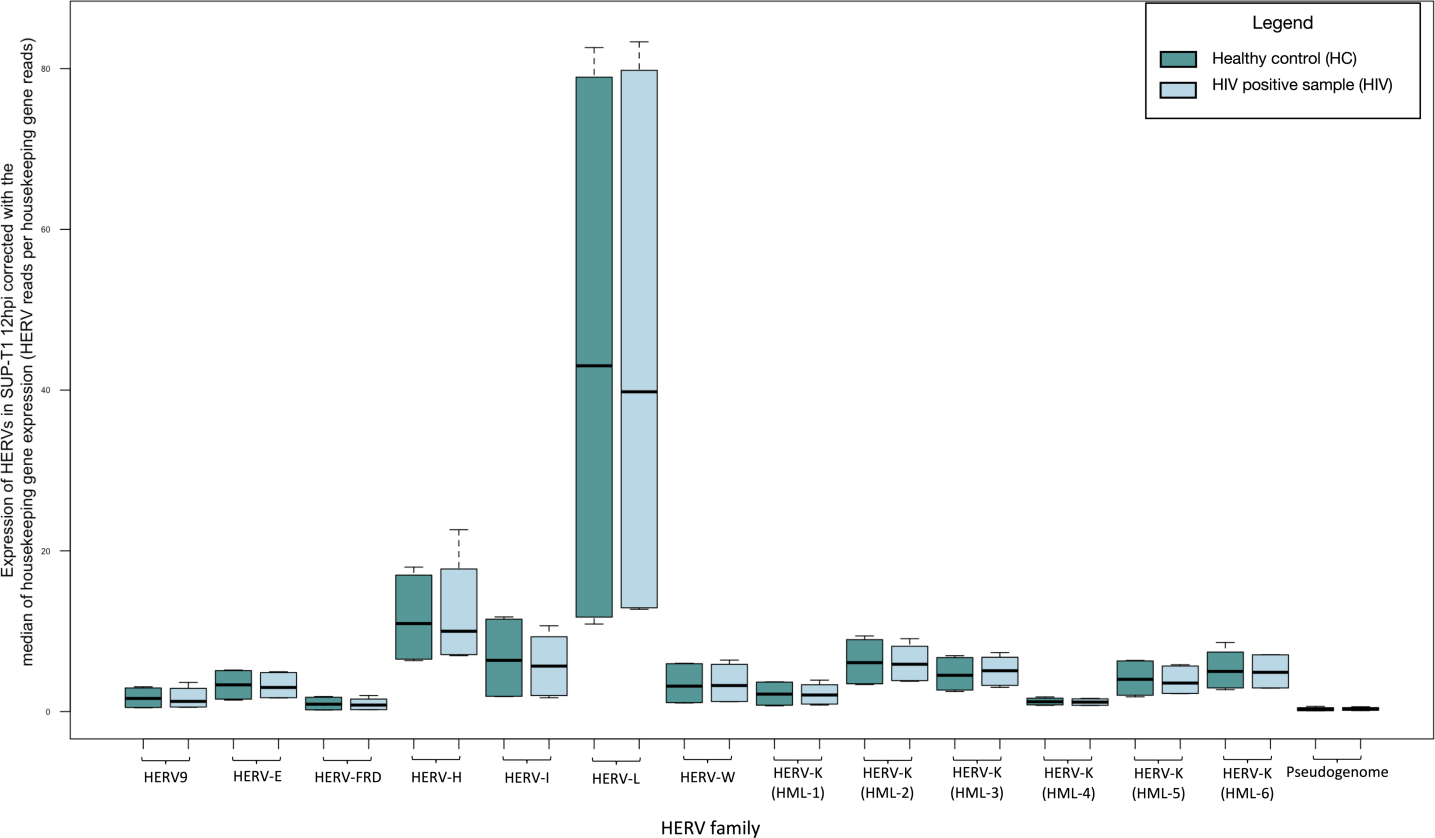
Transcription of HERVs in SUP-T1 cells 12 hours post infection with the HIV LAI strain compared to non-infected cells normalized by the median of the transcription of two housekeeping genes (succinate dehydrogenase A and hypoxanthine phosphoribosyltransferase 1) expressed as HERV reads per housekeeping gene reads.

**Fig 4:**
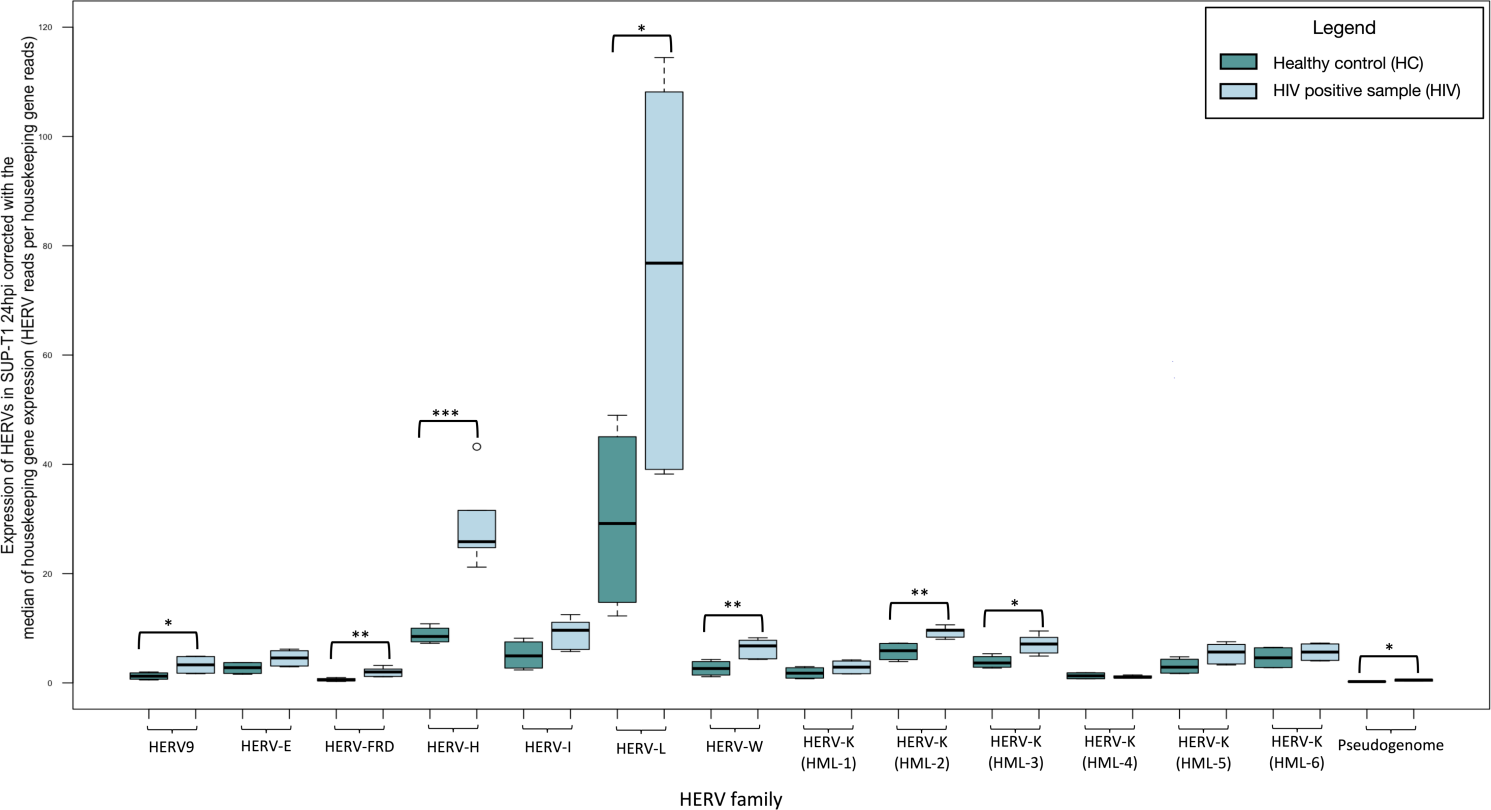
Transcription of HERVs in SUP-T1 cells 24 hours post infection with the HIV LAI strain compared to non-infected cells normalized by the median of the transcription of two housekeeping genes (succinate dehydrogenase A and hypoxanthine phosphoribosyltransferase 1) expressed as HERV reads per housekeeping gene reads. The significantly increased expression of HERVs is highlighted with black boxes. * indicates *P* < 0.05, ** indicate *P* < 0.01, and *** indicate *P* < 0.001.

At 12 hours, HIV LAI-infected SUP-T1 cells demonstrated no significant change in the expression of the HERVs examined compared to non-infected ones (Fig. 3; Table S2). At 24 hours post infection, we found increased expression of HERV-9 (fold change: 2.630, *P*-value: 0.020), HERV-FRD (fold change: 3.448, *P*-value: 0.003), HERV-H (fold change: 3.279, *P*-value <0.001), HERV-L (fold change: 2.528, *P*-value: 0.030), HERV-W (fold change: 2.392, *P*-value: 0.009), and two subgroups of HERV-K family HML-2 (fold change: 1.628, *P*-value: 0.005) and HML-2 (fold change: 1.843, *P*-value: 0.010) compared to non-infected samples. We recognized significant difference in the expression of the HK2 junction sites analyzed (fold change: 2.144, *P*-value: 0.015) (Fig. 4; Table 2).

**TABLE 2:** Transcription of HERVs in SUP-T1 cells 24 hours post HIV infection compared to non-infected normalized by the median of the transcription of two housekeeping genes (SDHA and HPRT1) expressed as HERV reads per housekeeping gene reads^*a*^

	*P*-value	Ratio
HERV-9	**0.020**	**2.630**
HERV-E	0.058	1.667
HERV-FRD	**0.003**	**3.448**
HERV-H	**<0.001**	**3.279**
HERV-I	0.051	1.785
HERV-L	**0.030**	**2.528**
HERV-W	**0.009**	**2.392**
HML-1	0.165	1.583
HML-2	**0.005**	**1.628**
HML-3	**0.010**	**1.843**
HML-4	0.668	0.838
HML-5	0.060	1.778
HML-6	0.330	1.221
Pseudogenome	**0.015**	**2.144**

^*a*^ The significant results are in bold. HPRT1, hypoxanthine phosphoribosyltransferase 1; SDHA, succinate dehydrogenase A; SUP-T1, Stanford University pediatric T cell line 1, from lymphoblastic lymphoma cells. Pseudogenome: To characterize the HK2 transcription at its integration sites, we created a “pseudogenome” based on the annotated “solo” LTR-start and -end coordinates of the HK2 sites.

### Significantly increased HERV expression in HIV LAI-infected MT4 T cell line compared to uninfected cells at 3 days post infection

**Fig 5:**
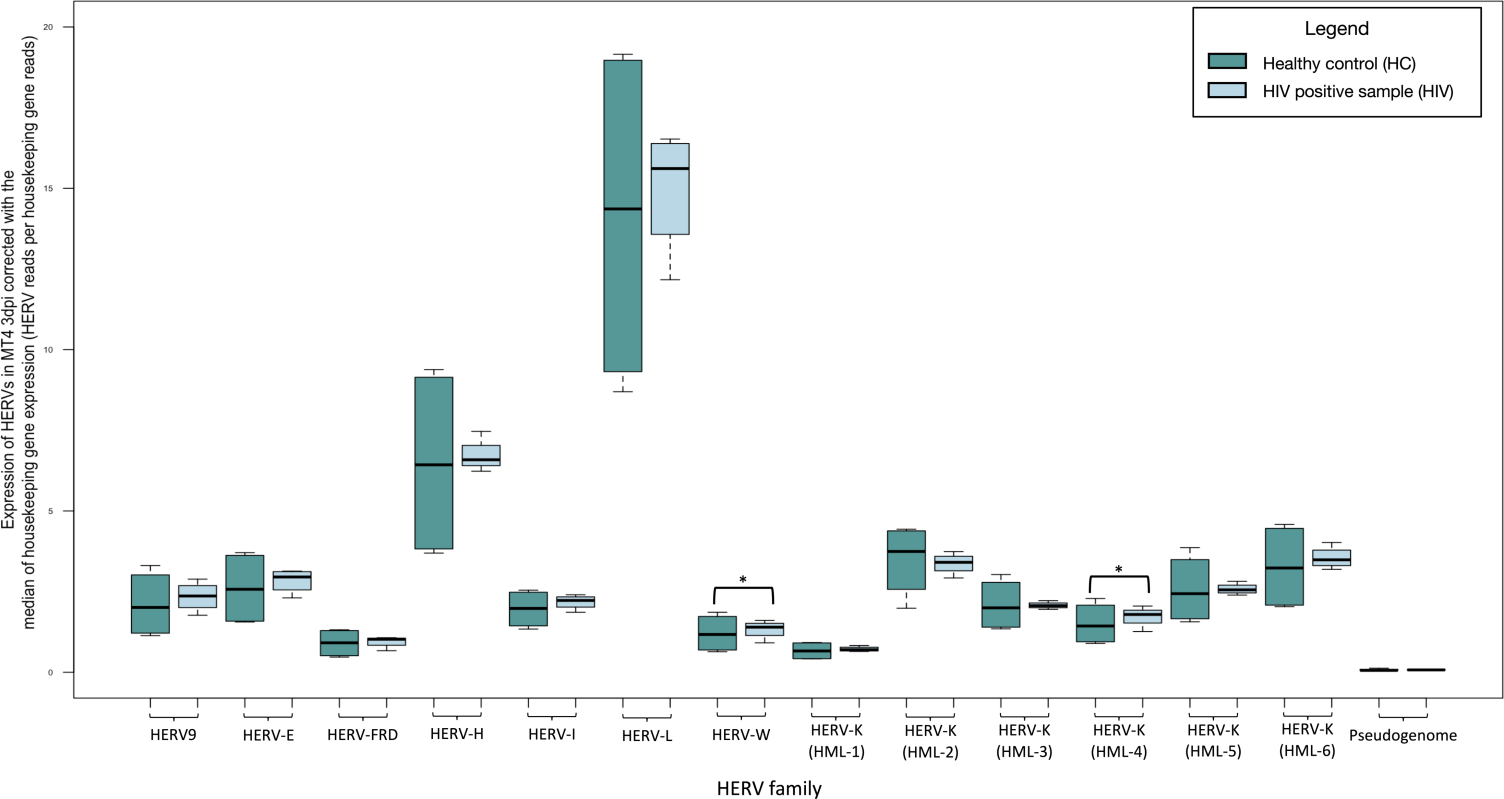
Transcription of HERVs in MT4 T cells 3 days post infection with the HIV LAI strain compared to non-infected normalized by the median of the transcription of two housekeeping genes (succinate dehydrogenase A and hypoxanthine phosphoribosyltransferase 1) expressed as HERV reads per housekeeping gene reads. The significantly increased expression of HERVs is highlighted with black boxes. * indicates *P* < 0.05.

In the MT4 T cell culture data sets, we found significantly increased expression of HERV-K (HML-4) (fold change: 1.576, *P*-value: 0.030), and HERV-W (fold change: 1.609, *P*-value: 0.046), at 3 days post infection compared to uninfected cells. We did not recognize significant differences in the expression of the HK2 junction sites (Fig. 5; Table S3).

### Significantly increased HERV-W expression after HIV infection of primary monocyte-derived macrophage (MDΜ) cells

**Fig 6:**
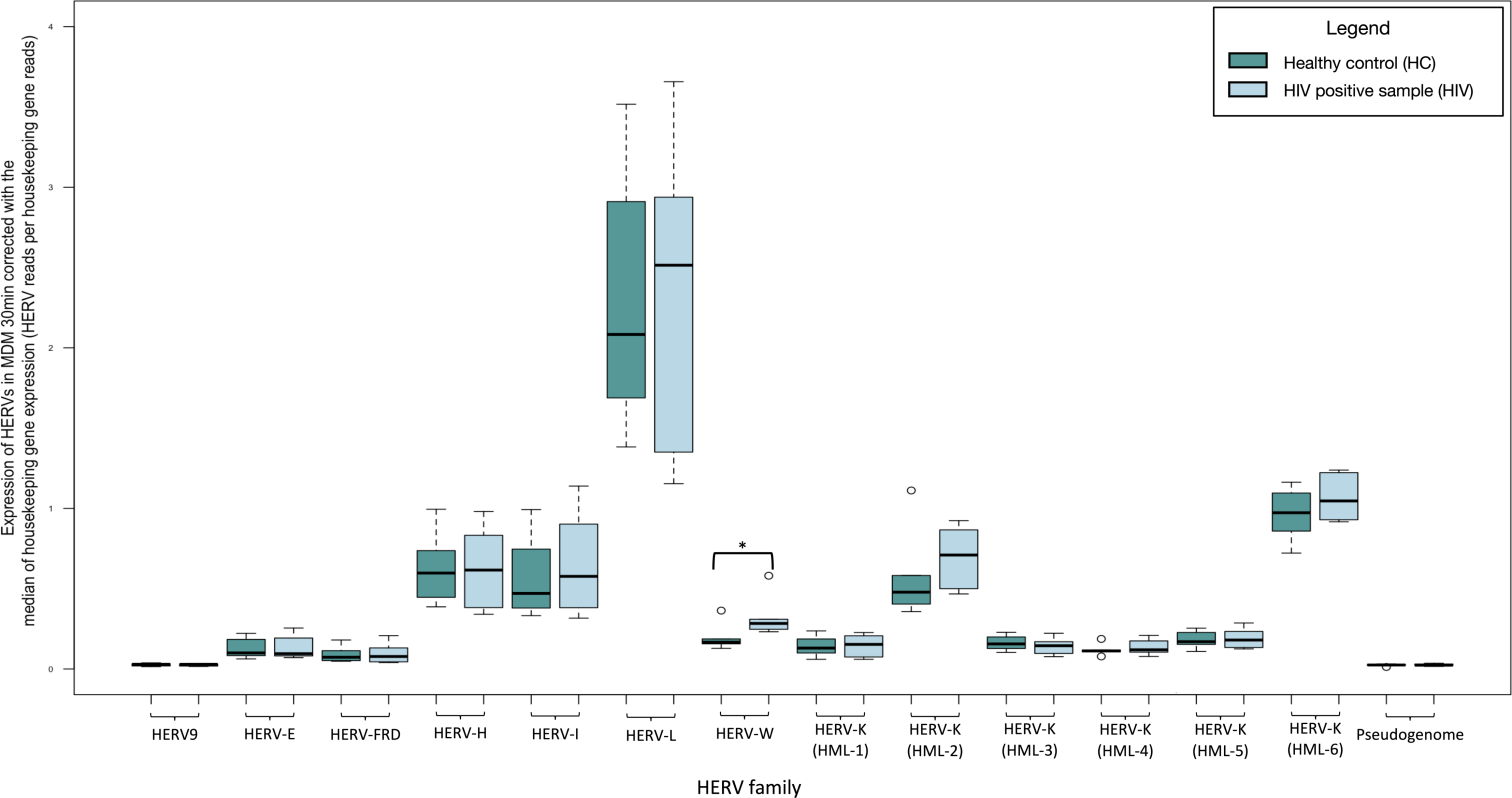
Transcription of HERVs in MDM 30 minutes post infection with HIV compared to non-infected normalized by the median of the transcription of two housekeeping genes (succinate dehydrogenase A and hypoxanthine phosphoribosyltransferase 1) expressed as HERV reads per housekeeping gene reads. The significantly increased expression of HERV-W is highlighted with black box. * indicates *P* < 0.05.

We recognized a significant change in the expression of the HERV-W family (fold change: 1.655, *P*-value: 0.028) examined at 30 minutes post infection in the HIV LAI-infected primary MDM cell cultures compared to their baseline expression before incubation with the virus. We did not recognize significant differences in the expression of the HK2 junction sites (Fig. 6; Table S4).

### No significant changes in expression of HERVs 16 hours after HIV LAI infection in monocyte cultures

**Fig 7:**
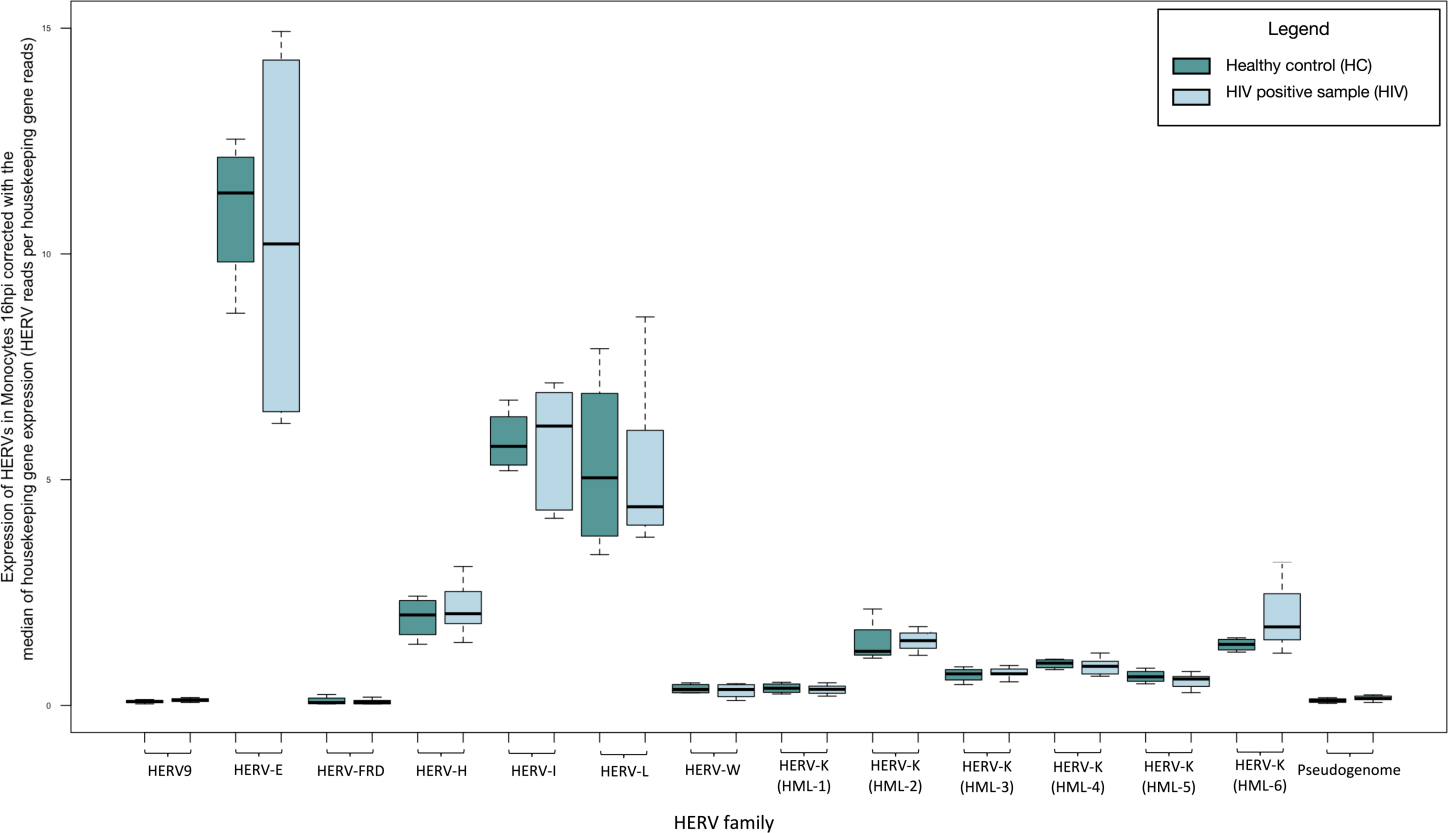
Transcription of HERVs in monocytes 16 hours post infection with HIV compared to non-infected cells normalized by the median of the transcription of two housekeeping genes (succinate dehydrogenase A and hypoxanthine phosphoribosyltransferase 1) expressed as HERV reads per housekeeping gene reads.

We did not recognize any significant increase in the expression of HERVs in HIV LAI-infected monocytes at 16 hours post infection compared to uninfected cells (Fig. 7; Table S5).

### Significantly decreased expression of HERV-K (HML-6) at 36 hours after HIV LAI infection in macrophages derived from healthy donors which does not persist at 6 days post infection

**Fig 8:**
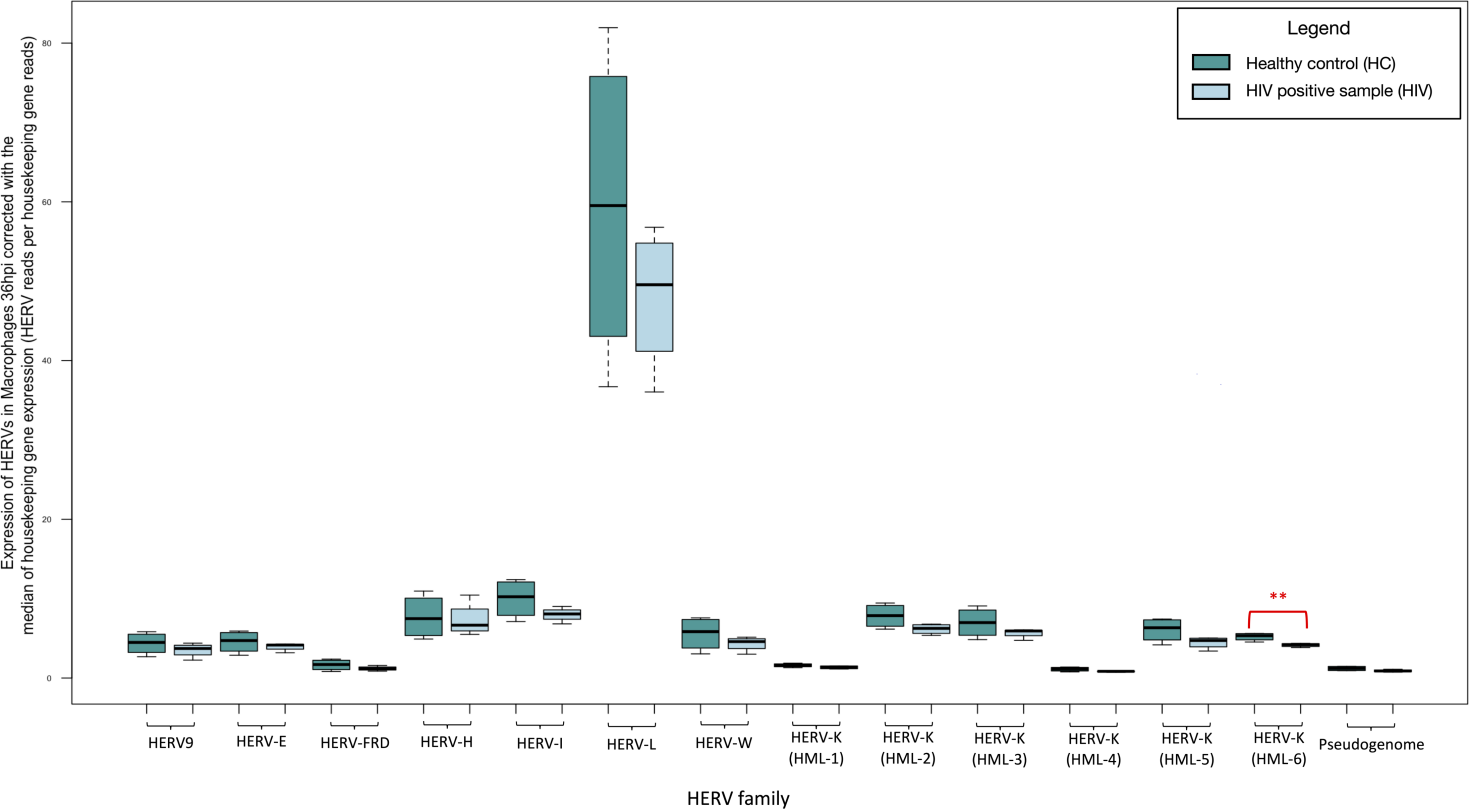
Transcription of HERVs in macrophages 36 hours post infection with HIV compared to non-infected normalized by the median of the transcription of two housekeeping genes (succinate dehydrogenase A and hypoxanthine phosphoribosyltransferase 1) expressed as HERV reads per housekeeping gene reads. The significantly decreased expression of HERV-K (HML-6) is highlighted with red box. ** indicate *P* < 0.01.

**Fig 9:**
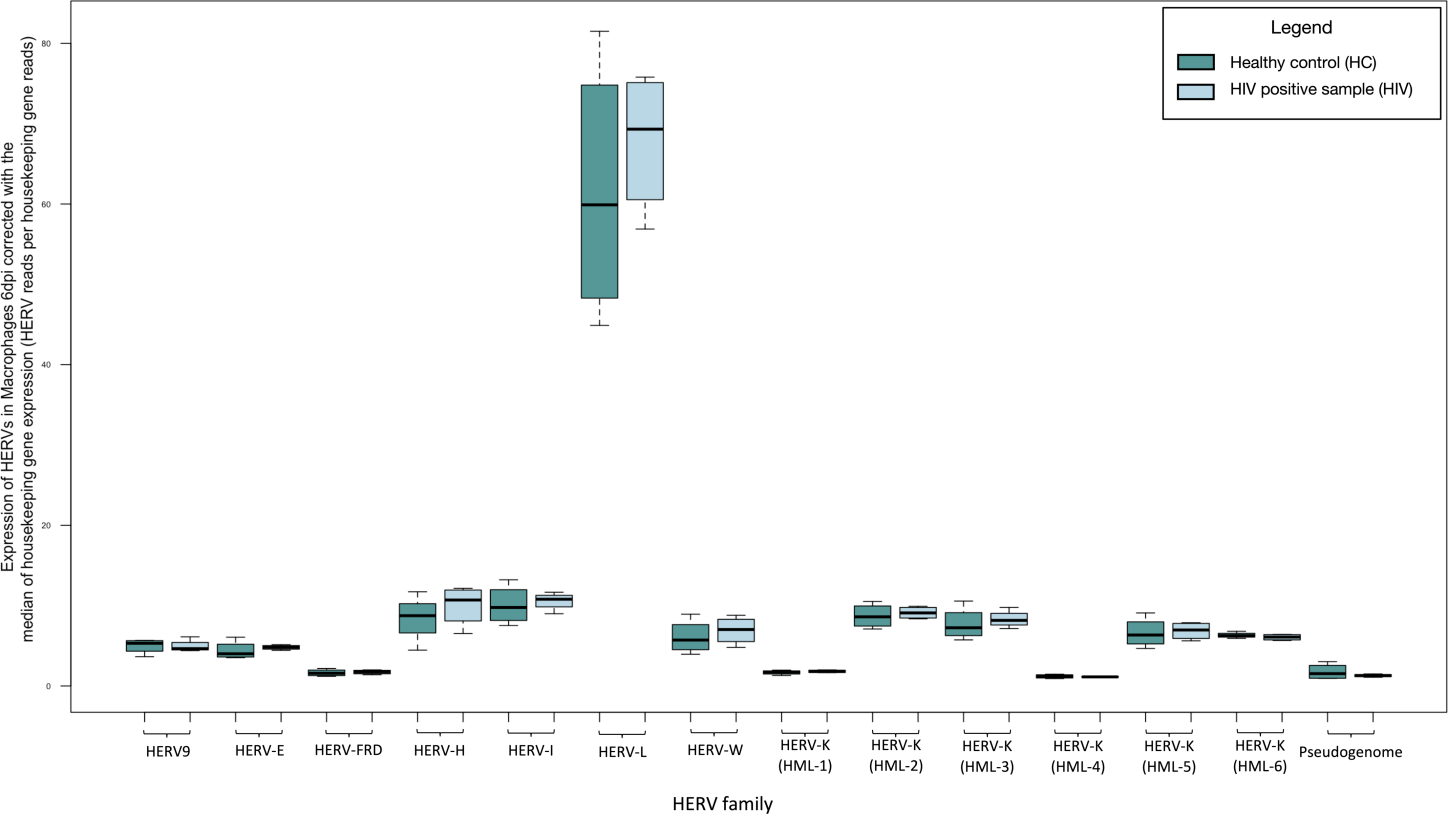
Transcription of HERVs in macrophages 6 days post infection with HIV compared to non-infected normalized by the median of the transcription of two housekeeping genes (succinate dehydrogenase A and hypoxanthine phosphoribosyltransferase 1) expressed as HERV reads per housekeeping gene reads.

We recognized significantly decreased expression of HERV-K (HML-6) (fold change: 0.798, *P*-value: 0.008) at 36 hours post infection compared to the mock infected cells at the same time point (Fig. 8; Table S6), which is not maintained in the 6 days post infection. (Fig. 9; Table S7). We did not recognize significant differences in the expression of the HK2 junction sites.

## DISCUSSION

In this work, we analyzed multiple datasets stemming from treatment-naïve HIV patients with viremia and patients receiving cART, primary cells derived from healthy donors, and cell lines *in vitro* infected with HIV and studied at different time points. We demonstrate a widespread HERV dysregulation in multiple cell lines, following HIV infection. We found significant deregulation in the expression of multiple HERV families in the PBMCs of HIV patients compared to PBMCs of healthy donors and more specifically, we demonstrate the decreased expression of HERV-H in the PBMCs of treatment-naïve HIV-infected individuals with viremia while a widespread increased expression of multiple HERV families is noted in HIV patients receiving cART.

Additionally, we also studied two types of T cell lines, namely SUP-T1 and MT4 T cells, where in both cases, we demonstrate an aberrant pattern of multiple HERV families expression at 24 hours and 3 days following *in vitro* HIV infection, respectively. In the latter two cases, the malignant burden on these cells as well as the additional retroviral burden in MT4 cell-line, which is derived from one human T-lymphotropic virus type 1 (HTLV-1)-positive patient with adult T cell leukemia and expresses the HTLV-1 Tax protein, might enhance the HERV deregulation (21).

We were also interested in studying the effect of *in vitro* HIV infection on cells of monocytic origin, more specifically monocyte-derived macrophages, macrophages derived from healthy donors, and monocyte cell cultures. Generally, a modest effect of HIV infection was observed in these cells of monocytic origin, with the increased expression of HERV-W in primary monocyte-derived macrophages soon after their exposure to HIV, while a decreased expression of HERV-K (HML-6) is present at 36 hours after HIV infection in macrophages derived from healthy donors which does not persist at 6 days.

Our results indicate that the type of cells infected by HIV as well as the timing of the HIV infection affect the levels of HERV expression. Most importantly, the time dependence on the HERV expression modification is apparent in the transient HERV-K (HML-6) downregulation at 36 hours after HIV infection in macrophages derived from healthy donors. These time-dependent differences can be explained in the frames of the generalized cellular gene expression deregulation that begins soon after the HIV infection in cells and takes place in a time-dependent manner as has been described in proteomic studies and by the time-dependent changes in the phosphorylation signatures after the HIV infection (22). These phosphorylation-signaling signatures were efficient to indicate the latently HIV-infected T cells as opposed to uninfected ones and were functionally relevant in the cellular death process, as they activated the p38 and c-Jun N-terminal kinase (JNK) signaling in the latently HIV-infected cells (22). Interestingly, the deregulated HERV expression throughout the course of HIV infection can apply as a plausible explanation behind these altered phosphorylation signaling, as HERV-K was found to alter the JNK kinase pathway in pancreatic cancer which enhances the tumorigenic process (23).

One of the most extensively studied intraviral interaction between HERVs and HIV regards that between HK2 and HIV, which indicates the HIV-mediated HK2 transactivation (18). More specifically, the HIV Tat (24) and Rev (25 – 29) proteins have been described to induce the increment of HK2 expression. On the other hand, HERV-K Gag leads to the reduction of HIV-1 replication, viral particle release, and infectivity (19), and HERV Env proteins are recognized as immunogenic exogenous antigens leading to innate and adaptive immune responses (20). HIV-infected individuals appear to develop anti-HK2 Env transmembrane IgM during the stage of the HIV-1 viremia (30) and HERV-K specific T cells, which can recognize HIV-1-, HIV-2-, and simian immunodeficiency virus-infected cells due to the expression of the viral infectivity factor (Vif) (31). One main role of the HIV Vif is to inhibit APOBEC deaminases (32), which also exert control over HERV expression; thus, APOBEC inhibition facilitates the increase in the expression of HERV proteins and, consequently, antiviral immune responses are triggered (33). In concordance with other groups that have demonstrated the increased expression of HK2 in the frameworks of HIV infection (28, 34), we also report significantly increased expression of HK2 in the PBMCs of HIV-infected patients under cART compared to healthy individuals and, also, following the infection with HIV of SUP-T1 cell cultures. Notably in the latter case, we report increased transcription at the HML-2-host junction sites, a fact which indicates the possibility throughout transcription at these sites in this case.

Interestingly, we also found a significantly increased expression of HERV-W in the PBMCs of HIV-infected patients compared to healthy donors, HIV-infected SUP-T1 and MT4 cell-lines, and monocyte-derived macrophages compared to non-infected cells. These results demonstrate clinical interest, since the HERV-W Envelope protein (syncytin-1) is the most striking example of HERV exaptation due to its critical role in the cellular fusion process, effective placental development, and protection of the developing embryo during the pregnancy (35). Recent data suggest that during HIV-1 infection, however, syncytin-1 favors the fusion of HIV-infected T cells and non-infected placental trophoblast cells, hence enhancing viral transmission (36), changing viral tropism toward trophoblast cells (36), and establishing HIV reservoirs in tissues, where antiretroviral therapy (ART) may demonstrate reduced bioavailability (36). Additionally, increased expression of HERV-9 occurred in the MT4 cell line culture following its infection with HIV, in the data sets included in the present analysis. HERV-9-derived LTR12C, which is located upstream of the interferon-inducible genes guanylate-binding proteins (GBPs), GBP2 and GBP5, was recently found to be activated. These genes participate in the antiviral immune mechanisms and LTR12C seems to have been under evolutionary exaptation to enhance the antiretroviral immunity following infection (37).

Multiple mechanisms have been proposed for the modification of HERV transcription, most notably HERV-K, and protein expression by HIV-1, the majority of which appear to be mediated by retroviral proteins. One important player in the upregulation of HERV loci transcription in the setting of HIV infection appears to be the HIV-1 Tat protein. More specifically, Tat leads to the activation of nuclear factor kappa-light-chain-enhancer of activated B cells (NF-κB) and Nuclear factor of activated T-cells (NF-AT) transcription factors, which directly function on the HK2 LTR promoters (28). The effect of the Tat-mediated HK2 activation is more pronounced in the cells, that HIV-1 targets, such as lymphocytes and monocytes, and HK2 gag as well as the oncogenes, np9 and rec are the most upregulated HK2 transcripts in Jurkat T cells and primary lymphocytes (28). Additionally, Tat was found to increase the HERV-K expression through modifications in the heterochromatin structure (19). Furthermore, transcription factors such as NF-AT and activator protein 1 (AP-1) are possible activators of multiple HERV families, the transcription of which is deregulated throughout the HIV-infection in T cells, both at the *de novo* infection stage as well as in chronically infected T cells (17). Regarding the levels of HERV-K protein expression, the nuclear export of HERV-K (type 2) and HIV-1, and thus their cytoplasmic availability, is facilitated by the binding of the retroviral proteins Rec and Rev to a specific region on their messenger RNAs (mRNAs), Rec-response element (RcRE), and Rev response element (RRE), respectively. At least partially, the increased production of HERV-K proteins in HIV-infected patients can be attributed to increased export of HERV-K mRNAs resulting from the structural homology between the Rev and the HERV-K Rec (27).

The main limitation of our study is that since we analyzed publicly available data sets, we could not have access directly to the demographics and exact medical history of the cohorts studied, and also we were not able to measure the HERV expression in the studied cell cultures at multiple time points consistently. Furthermore, most of the analyzed data sets stem from *in vitro* HIV infection of various cell cultures, including the SUP-T1 and MT4 cell lines, and monocyte-derived macrophages, macrophages from healthy donors, and monocyte cell cultures. Despite the valuable inferences made from *in vitro* studies, the exact intricate conditions during HIV infection *in vivo* are not possible to be simulated, such as the immune responses to the HIV viral triggers, the effect of HERV activation on the interferonogenic immune responses (20, 29), and a further level of control exerted by the cellular environment, as the remodeling of cellular chromatin and the changes in methylation patterns (38). Additionally, our small sample size may have been an obstacle in the recognition of additional potential significant dysregulations.

However, we have been able to demonstrate biologically significant results regarding the effect of HIV infection on the expression of HERVs in PBMCs, T cell, and monocytic cell cultures, consistent with the existing literature.

Finally, in this work, we have been able to demonstrate that HIV infection affects the expression in multiple HERV families in the PBMCs of HIV-infected patients, cell lines derived by T cells, and modest modification of their expression is detected in cell cultures of monocytic origin. These results indicate that further evaluation of the effect of HIV infection on the endogenous retroviral regulation in the affected cells and tissues and their subsequent implications during HIV infection is needed.

## MATERIALS AND METHODS

To perform this analysis, we used publicly available data sets from the National Center for Biotechnology Information (NCBI) Sequence Read Archive (https://www.ncbi.nlm.nih.gov/sra).

### Data sets

We compared the HERV expression in PBMCs from treatment-naïve HIV patients with viremia and healthy controls from Bioproject PRJNA420459 (Illumina HiSeq 2500, paired-end reads, 126 base length); we selected a total of 13 healthy controls (SRR6333695, SRR6333706, SRR6333707, SRR6333708, SRR6333709, SRR6333710, SRR6333711, SRR6333712, SRR6333713, SRR6333714, SRR6333715, SRR6333720, SRR6333729) and eight treatment-naïve HIV-1-infected patients (SRR6333716, SRR6333717, SRR6333718, SRR6333719, SRR6333729, SRR6333732, SRR6333733, SRR6333734).

For the comparison of HERV family expression in the PMBCs from patients under cART and healthy controls, the data sets on Bioproject PRJEB34025 (Illumina HiSeq 2500, single-end reads, 50 base length) were analyzed. Of these, 22 data sets correspond to HIV-positive individuals under antiretroviral treatment and 15 are healthy controls.

For the comparison between the levels of HERV expression between cell cultures of SUP-T1, from lymphoblastic lymphoma cells (SUP-T1 cells) infected with HIV LAI strain (derived from patient LAI, 21) and mock infection at 12 and 24 hours after infection compared to uninfected cell cultures, Bioprojects PRJNA167037 (Illumina Genome Analyzer IIx, single-end reads of 75 base length) (39) and PRJNA234297 (Illumina HiSeq 2000, paired-end reads 180 base length) (40) were analyzed. In these two datasets, we selected a total of six HIV LAI-infected data sets and six uninfected data sets at 12 hours post infection, while at 24 hours post infection, we analyzed four uninfected data sets and six HIV LAI-infected; all of these data sets stemmed from cell cultures that had not been treated with any compound so we could study the direct effects of HIV infection (PRJNA167037: SRR497705, SRR497706, SRR497707, SRR497708, SRR497709, SRR497699, SRR497700, SRR497701, SRR497702, SRR497703, SRR497704-PRJNA234297: SRR1106195, SRR1106196, SRR1106197,SRR1106198, SRR1106199, SRR1106189, SRR1106190, SRR1106191, SRR1106192, SRR1106193, SRR1106194).

For the comparison between the HERV level expression between cell cultures of MT4 T cells, four data sets were infected by HIV LAI and four were from mock infection, Bioproject PRJNA298743 (Illumina HiSeq 2500, single-end reads, 50 base length) was analyzed (41).

For the comparison between the levels of HERV expression in primary MDMs at baseline and at 4 days after a 30-minute treatment of these cells with HIV, Bioproject PRJNA403862 (Illumina HiSeq 3000, single-end reads of 50 base length) was analyzed; in this data set, we had six HIV-infected data sets and six uninfected data sets (42).

For the comparison between the expression of the studied HERV families in cell cultures of primary macrophages from four healthy donors infected *in vitro* with HIV and mock infection analyzed 36 hours post infection (Donor 1: SRR15178990, SRR15178991, SRR15178992, SRR15178993, SRR15178998, SRR15178999, SRR15179000, SRR15179001; Donor 2: SRR15179014, SRR15179015, SRR15179016, SRR15179017, SRR15179022, SRR15179023, SRR15179024, SRR15179025; Donor 3: SRR15179038, SRR15179039, SRR15179040, SRR15179041, SRR15179046, SRR15179047, SRR15179048, SRR15179049 and Donor 4: SRR15179062, SRR15179063, SRR15179064, SRR15179065, SRR15179070, SRR15179071, SRR15179072, SRR15179073) and 6 days post infection (Donor 1: SRR15178994, SRR15178995, SRR15178996, SRR15178997, SRR15179002, SRR15179003, SRR15179004, SRR15179005; Donor 2: SRR15179018, SRR15179019, SRR15179020, SRR15179021, SRR15179026, SRR15179027, SRR15179028, SRR15179029; Donor 3: SRR15179042, SRR15179043, SRR15179044, SRR15179045, SRR15179050, SRR15179051, SRR15179052, SRR15179053; Donor 4: SRR15179066, SRR15179067, SRR15179068, SRR15179069, SRR15179074, SRR15179075, SRR15179076, SRR15179077), the control cells in this case have been obtained by a mock infection of the donor cells and are analyzed again after 36 hours (Donor 1: SRR15179006, SRR15179007, SRR15179008, SRR15179009; Donor 2: SRR15179030, SRR15179031, SRR15179032, SRR15179033; Donor 3: SRR15179054, SRR15179055, SRR15179056, SRR15179057; Donor 4: SRR15179078, SRR15179079, SRR15179080, SRR15179081) and 6 days post infection (Donor 1: SRR15179010, SRR15179011, SRR15179012, SRR15179013; Donor 2: SRR15179034, SRR15179035, SRR15179036, SRR15179037; Donor 3: SRR15179058, SRR15179059, SRR15179060, SRR15179061; Donor 4: SRR15179082, SRR15179083, SRR15179084, SRR15179085). Since we analyzed biological replicates, we calculated the average of expression of each donor and made the comparison between mock infected and infected cell for each time point. Bioproject PRJNA747301 (Illumina HiSeq 1000, paired-end reads of 101 base length) was used for these comparisons (43).

We also compared the levels of HERV expression in cell cultures of monocytes from healthy donors *in vitro* infected with HIV LAI strain and uninfected cells, at 16 hours post infection. From Bioproject PRJNA762357 (Illumina HiSeq 2500, paired-end reads, 300 base length), we used the untreated samples (i.e., not treated with lipopolysaccharide or other compounds) for the comparison between HERV family expression levels at baseline and at 16 hours after infection (non-infected: SRR15845863, SRR15845864, SRR15845878, and SRR15845879; infected: SRR15845865, SRR15845866, SRR15845867, SRR15845868, SRR15845890, SRR15845891, SRR15845901, and SRR15845902).

### Family-wide analysis

We analyzed the expression of HERVs at a family level. The first step was to perform the alignment against hg19 using Bowtie2 (44) with the default settings applying the features for the single- or paired-end layout, followed by the Samtools view commands to set the quality of the reads above 20, and then to sort and index the produced files (45).

We used the bedtools multicov command (46) to be able to quantify the expression of each family of HERVs by isolating reads aligned at HERV loci. We set the -f option to include reads that are aligned at HERV loci at least at 80% of their length. For the calculation of the -f option in bedtools multicov command, we considered a length of 10,000 bp for HML-1, HML-2, HML-4, HERV-W, HERV-L, HERV-E, and HERV-I, 9,000 bp for HML-3, HML- 5, and HML-6 and HERV-FRD, 12,000 bp for HERV-9, and 17,000 bp for HERV-H (47), and then we divided the 80% of read length in each data set to the length of the virus.

We analyzed 13 HERV families, based on their annotated coordinates: HERV-9, HERV-E, HERV-FRD, HERV-H, HERV-I, HERV-L, HERV-W, HERV-K (HML-1), HK2 (3), HERV-K (HML-3), HERV-K (HML-4), HERV-K (HML-5), and HERV-K (HML-6) (48 – 50).

In order to normalize our results, we performed the alignment with two housekeeping genes hypoxanthine phosphoribosyltransferase 1 and succinate dehydrogenase A.

We calculated the sum of the total transcripts corresponding to each virus, and we divided this number to the reads corresponding to each of the housekeeping gene. To obtain the normalized expression, we calculated the average of the two separate normalizations, with each of the genes.

### Transcription running over HK2 integrations analysis

To characterize the HK2 transcription at its integration sites, we aimed to detect the expression at the HK2-host junction sites. Hence, we created a “pseudogenome,” based on the annotated “solo” LTR-start and -end coordinates of the HK2 (3) and we isolated the junction sites between human genome and HK2 and their flanking regions at a length depending on the features of the data sets analyzed. To achieve this, we calculated 66% of the length of the reads of the data sets analyzed, and we used this length to determine the width of the flanking region per case on each side of the LTR-start and -end genomic positions. Thus, the genomic coordinates we isolated included the virus-human junctions.

Bedtools getfasta command (46) was used to extract the exact sequences in the human genome and these sequences were indexed using Bowtie2. We aligned the analyzed data sets against the “pseudogenome” using Bowtie2 (44) with default settings, then we followed the same workflow as described previously. The normalization process applied is the same as previously described.

### Statistical analysis

We used R Studio [RStudio Team (2022). RStudio: Integrated Development Environment for R. RStudio, PBC, Boston, MA URL http://www.rstudio.com/]. We used the natural logarithm transformed values of our numeric results to apply parametric tests. The independent sample Student’s *t*-test, equal variances not assumed (Welsh test), has been performed, to compare the levels of HERV expression as calculated in the family-wide analysis as well as for the expression at the HK2 junction sites (“pseudogenome” analysis). Statistical significance level was set at *P* < 0.05.

## Data Availability

The datasets analyzed during the current study are publicly available on NCBI Sequence Read Archive (SRA). Data sets PRJEB34025, PRJNA420459, PRJNA167037, PRJNA234297, PRJNA298743, PRJNA403862, PRJNA747301 were analyzed. From datasetdata set PRJNA762357, accession numbers: SRR15845863, SRR15845864, SRR15845878, SRR15845879, SRR15845865, SRR15845866, SRR15845867, SRR15845868, SRR15845890, SRR15845891, SRR15845901 and SRR15845902 were included in this analysis.

## SUPPLEMENTAL MATERIAL

Supplemental Tables spectrum.01379-23-s0001.docx Supplemental Material-Supplemental tables.

ASM does not own the copyrights to Supplemental Material that may be linked to, or accessed through, an article. The authors have granted ASM a non-exclusive, world-wide license to publish the Supplemental Material files. Please contact the corresponding author directly for reuse.
