# Supplementary material for: The interaction of human immunodeficiency virus-1 and human endogenous retroviruses in patients (primary cell cultures) and cell line models
Source: Microbiol Spectr. 2023 Oct 9;11(6):e01379-23. doi: 10.1128/spectrum.01379-23 (PMC10715072; doi:10.1128/spectrum.01379-23)
Supplement: Supplemental Tables — Supplemental Material-Supplemental tables. [file spectrum.01379-23-s0001.docx]

**The interaction of Human Immunodeficiency Virus-1 and Human Endogenous Retroviruses in Patients** **(primary cell cultures) and Cell-line models**

Federica Mantovani^*^, Konstantina Kitsou^*^, Dimitrios Paraskevis, Pagona Lagiou, Gkikas Magiorkinis^#^

Supplemental Material

| **Table 1:** Transcription of Human Endogenous Retroviruses (HERVs) in PBMCs cells from treatment naïve patients compared to healthy controls normalized by the median of the transcription of two housekeeping genes (SDHA and HPRT1) expressed as HERV reads per housekeeping gene reads. |
| --- |

|  | p-value | Ratio |
| --- | --- | --- |
| HERV-9 | 0.117 | 0.543 |
| HERV-E | 0.586 | 1.049 |
| HERV-FRD | 0.082 | 0.601 |
| HERV-H | **0.036** | **0.623** |
| HERV-I | 0.488 | 1.061 |
| HERV-L | 0.060 | 0.707 |
| HERV-W | 0.385 | 0.733 |
| HML-1 | 0.705 | 0.867 |
| HML-2 | 0.087 | 0.732 |
| HML-3 | 0.779 | 0.869 |
| HML-4 | 0.051 | 0.770 |
| HML-5 | 0.451 | 0.800 |
| HML-6 | 0.158 | 0.692 |
| Pseudogenome | 0.082 | 0.706 |
| The significant results are highlighted. Abbreviations: HERVs (Human Endogenous Retroviruses), HIV (Human immunodeficiency virus), SDHA (Succinate Dehydrogenase A), HPRT1 (Hypoxanthine Phosphoribosyltransferase 1)  Pseudogenome: To characterise the HK2 transcription at its integration sites, we created a “pseudogenome”, based on the annotated “solo” LTR-start and -end coordinates of the HK2 sites. | | |

| **Table 2:** Transcription levels of Human Endogenous Retroviruses (HERVs) in SUP-T1 cells infected with HIV LAI strain compared to non-infected 12 hours post infection, normalized by the median of the transcription of two housekeeping genes (SDHA and HPRT1) expressed as HERV reads per housekeeping gene reads. | | |
| --- | --- | --- |
|  | p-value | Ratio |
| HERV-9 | 0.968 | 0.989 |
| HERV-E | 0.951 | 0.967 |
| HERV-FRD | 0.974 | 0.958 |
| HERV-H | 0.849 | 1.067 |
| HERV-I | 0.903 | 0.880 |
| HERV-L | 0.938 | 0.993 |
| HERV-W | 0.892 | 1.037 |
| HML-1 | 0.914 | 0.993 |
| HML-2 | 0.962 | 0.980 |
| HML-3 | 0.662 | 1.095 |
| HML-4 | 0.828 | 0.945 |
| HML-5 | 0.999 | 0.940 |
| HML-6 | 0.910 | 0.940 |
| Pseudogenome | 0.716 | 1.082 |
| Abbreviations: HERVs (Human Endogenous Retroviruses), SUP-T1 (Stanford University Paediatric T-Cell Line 1, From Lymphoblastic Lymphoma Cells), HIV (Human Immunodeficiency virus), SDHA (Succinate Dehydrogenase A), HPRT1 (Hypoxanthine Phosphoribosyltransferase 1).  Pseudogenome: To characterise the HK2 transcription at its integration sites, we created a “pseudogenome”, based on the annotated “solo” LTR-start and -end coordinates of the HK2 sites. | | |

| **Table 3:** Transcription of Human Endogenous Retroviruses (HERVs) in MT4 T-cells infected with HIV compared to non-infected normalized by the median of the transcription of two housekeeping genes (SDHA and HPRT1) expressed as HERV reads per housekeeping gene reads. |
| --- |

|  | p-value | Ratio |
| --- | --- | --- |
| HERV-9 | 0.056 | 1.578 |
| HERV-E | 0.093 | 1.415 |
| HERV-FRD | 0.080 | 1.495 |
| HERV-H | 0.284 | 1.235 |
| HERV-I | 0.066 | 1.314 |
| HERV-L | 0.114 | 1.332 |
| HERV-W | **0.046** | **1.609** |
| HML-1 | 0.172 | 1.299 |
| HML-2 | 0.274 | 1.191 |
| HML-3 | 0.445 | 1.116 |
| HML-4 | **0.030** | **1.576** |
| HML-5 | 0.364 | 1.154 |
| HML-6 | 0.179 | 1.285 |
| Pseudogenome | 0.129 | 1.400 |
| The significant results are highlighted. Abbreviations: HERVs (Human Endogenous Retroviruses), HIV (Human immunodeficiency virus), SDHA (Succinate Dehydrogenase A), HPRT1 (Hypoxanthine Phosphoribosyltransferase 1)  Pseudogenome: To characterise the HK2 transcription at its integration sites, we created a “pseudogenome”, based on the annotated “solo” LTR-start and -end coordinates of the HK2 sites. | | |

| **Table 4:** Transcription levels of Human Endogenous Retroviruses (HERVs) in MDMs infected with HIV compared to non-infected normalized by the median of the transcription of two housekeeping genes (SDHA and HPRT1) expressed as HERV reads per housekeeping gene reads. | | |
| --- | --- | --- |
|  | p-value | Ratio |
| HERV-9 | 0.915 | 0.967 |
| HERV-E | 0.913 | 1.051 |
| HERV-FRD | 0.974 | 1.069 |
| HERV-H | 0.947 | 1.002 |
| HERV-I | 0.676 | 1.148 |
| HERV-L | 0.990 | 1.034 |
| HERV-W | **0.028** | **1.655** |
| HML-1 | 0.992 | 1.037 |
| HML-2 | 0.270 | 1.224 |
| HML-3 | 0.458 | 0.881 |
| HML-4 | 0.608 | 1.127 |
| HML-5 | 0.818 | 1.051 |
| HML-6 | 0.255 | 1.106 |
| Pseudogenome | 0.775 | 1.048 |
| The significant results are highlighted in bold when there is up-regulation, the bold and italic font indicates the downregulation. The asterisks are used to indicate the grade of significance.  Abbreviations: HERVs (Human Endogenous Retroviruses), MDM (Monocytes-Derived Macrophages), HIV (Human Immunodeficiency virus), SDHA (Succinate Dehydrogenase A), HPRT1 (Hypoxanthine Phosphoribosyltransferase 1).  Pseudogenome: To characterise the HK2 transcription at its integration sites, we created a “pseudogenome”, based on the annotated “solo” LTR-start and -end coordinates of the HK2 sites. | | |

| **Table 5:** Transcription levels of Human Endogenous Retroviruses (HERVs) in monocytes infected with HIV LAI strain for 16 hours and compared to non-infected normalized by the median of the transcription of two housekeeping genes (SDHA and HPRT1) expressed as HERV reads per housekeeping gene reads. | | |
| --- | --- | --- |
|  | p-value | Ratio |
| HERV-9 | 0.166 | 1.391 |
| HERV-E | 0.598 | 0.947 |
| HERV-FRD | 0.874 | 0.814 |
| HERV-H | 0.568 | 1.105 |
| HERV-I | 0.795 | 0.985 |
| HERV-L | 0.905 | 0.968 |
| HERV-W | 0.489 | 0.877 |
| HML-1 | 0.661 | 0.921 |
| HML-2 | 0.675 | 1.197 |
| HML-3 | 0.548 | 1.065 |
| HML-4 | 0.483 | 0.933 |
| HML-5 | 0.317 | 0.842 |
| HML-6 | 0.106 | 1.455 |
| Pseudogenome | 0.148 | 1.483 |
| Abbreviations: HERVs (Human Endogenous Retroviruses), HIV (Human Immunodeficiency virus), SDHA (Succinate Dehydrogenase A), HPRT1 (Hypoxanthine Phosphoribosyltransferase 1).  Pseudogenome: To characterise the HK2 transcription at its integration sites, we created a “pseudogenome”, based on the annotated “solo” LTR-start and -end coordinates of the HK2 sites. | | |

| **Table 6**: Transcription levels of Human Endogenous Retroviruses (HERVs) in macrophages from healthy donors infected with HIV for 36 hours and compared to non-infected normalized by the median of the transcription of two housekeeping genes (SDHA and HPRT1) expressed as HERV reads per housekeeping gene reads. | | |
| --- | --- | --- |
|  | p-value | Ratio |
| HERV-9 | 0.375 | 0.806 |
| HERV-E | 0.531 | 0.865 |
| HERV-FRD | 0.345 | 0.726 |
| HERV-H | 0.838 | 0.949 |
| HERV-I | 0.181 | 0.800 |
| HERV-L | 0.367 | 0.808 |
| HERV-W | 0.378 | 0.777 |
| HML-1 | 0.130 | 0.840 |
| HML-2 | 0.071 | 0.788 |
| HML-3 | 0.231 | 0.813 |
| HML-4 | 0.078 | 0.748 |
| HML-5 | 0.101 | 0.737 |
| HML-6 | ***0.008*** | ***0.798*** |
| Pseudogenome | 0.095 | 0.750 |
| The significant results are highlighted in bold when there is up-regulation, the bold and italic indicates the downregulation. The asterisk is used to indicate the grade of significance.  Abbreviations: HERVs (Human Endogenous Retroviruses), HIV (Human Immunodeficiency virus), SDHA (Succinate Dehydrogenase A), HPRT1 (Hypoxanthine Phosphoribosyltransferase 1)  Pseudogenome: To characterise the HK2 transcription at its integration sites, we created a “pseudogenome”, based on the annotated “solo” LTR-start and -end coordinates of the HK2 sites. | | |

| **Table 7:** Transcription levels of Human Endogenous Retroviruses (HERVs) in macrophages from healthy donors infected with HIV for 6 days and compared to non-infected normalized by the median of the transcription of two housekeeping genes (SDHA and HPRT1) expressed as HERV reads per housekeeping gene reads. | | |
| --- | --- | --- |
|  | p-value | Ratio |
| HERV-9 | 0.851 | 0.993 |
| HERV-E | 0.489 | 1.092 |
| HERV-FRD | 0.714 | 1.057 |
| HERV-H | 0.467 | 1.190 |
| HERV-I | 0.710 | 1.049 |
| HERV-L | 0.509 | 1.102 |
| HERV-W | 0.575 | 1.137 |
| HML-1 | 0.504 | 1.081 |
| HML-2 | 0.686 | 1.047 |
| HML-3 | 0.625 | 1.081 |
| HML-4 | 0.526 | 0.944 |
| HML-5 | 0.824 | 1.037 |
| HML-6 | 0.186 | 0.960 |
| Pseudogenome | 0.637 | 0.734 |
| Abbreviations: HERVs (Human Endogenous Retroviruses), HIV (Human Immunodeficiency virus), SDHA (Succinate Dehydrogenase A), HPRT1 (Hypoxanthine Phosphoribosyltransferase 1)  Pseudogenome: To characterise the HK2 transcription at its integration sites, we created a “pseudogenome”, based on the annotated “solo” LTR-start and -end coordinates of the HK2 sites. | | |
